# Incidence and predictors of surgical site infections following caesarean sections at Bugando Medical Centre, Mwanza, Tanzania

**DOI:** 10.1186/2047-2994-3-25

**Published:** 2014-08-11

**Authors:** Filbert J Mpogoro, Stephen E Mshana, Mariam M Mirambo, Benson R Kidenya, Balthazar Gumodoka, Can Imirzalioglu

**Affiliations:** 1 Department of Obstetrics and Gynecology Weill School of Medicine, CUHAS-Bugando, Mwanza, Tanzania; 2 Department of Microbiology/Immunology Weill School of Medicine, CUHAS-Bugando, BOX 1464, Mwanza, Tanzania; 3 Department of Biochemistry and Molecular Biology, Weill School of Medicine, CUHAS-Bugando, Mwanza, Tanzania; 4 Institute of Medical Microbiology, Justus-Liebig-University of Giessen, Giessen, Germany

## Abstract

**Background:**

Surgical site infection (SSI) is the second most common infectious complication after urinary tract infection following a delivery by caesarean section (CS). At Bugando Medical Centre there has no study documenting the epidemiology of SSI after CS despite the large number of CSs performed and the relatively common occurrence of SSIs.

**Methods:**

This was a prospective cohort study involving pregnant women who underwent a CS between October 2011 and February 2012 at Bugando Medical Centre. A total of 345 pregnant women were enrolled. Preoperative, intraoperative and postoperative data were collected using a standardized questionnaire. Wound specimens were collected and processed as per standard operative procedures; and susceptibility testing was carried out using a disc diffusion technique. Data was analyzed using STATA version 11.

**Results:**

The overall cumulative incidence of SSI was 10.9% with an incidence rate of 37.5 per 10,000 people/day (95% CI, 26.8-52.4). The median time from CS to the development of SSI was 7 days (interquartile range [IQR] = 6–9 days). Six independent risk factors for post caesarean SSI as identified in this study by multivariate analysis are: hypertensive disorders of pregnancy (HR: 2.5; 95% CI, 1.1-5.6; *P =* 0.021), severe anaemia (HR: 3.8; 95% CI, 1.2-12.4, *P =* 0.028), surgical wound class III (HR: 2.4; 95% CI, 1.1-5.0; *P =* 0.021), multiple vaginal examinations (HR: 2.5; 95% CI, 1.2-5.1; *P =* 0.011), prolonged duration of operation (HR: 2.6; 95% CI, 1.2-5.5; *P =* 0.015) and an operation performed by an intern or junior doctor (HR: 4.0; 95% CI, 1.7-9.2; *P =* 0.001). *Staphylococcus aureus* was the most common organism (27.3%), followed by *Klebsiella pneumoniae* (22.7%). Patients with a SSI had a longer average hospital stay than those without a SSI (12.7 ± 6.9 vs. 4 ± 1.7; *P* < 0.0001) and the case fatality rate among patients with a SSI was 2.9%.

**Conclusion:**

SSIs are common among women undergoing CSs at Bugando Medical Centre. SSIs were commonly associated with multiple factors. Strategies to control these factors are urgently needed to control SSIs post CS at Bugando Medical Centre and other centres in developing countries.

## Background

Caesarean section (CS) is the most commonly performed major abdominal operations among women in both developed and developing countries [1]. Globally, the CS rate average is approximately 15% [1]; however there is a great variation in the rate of CSs in Latin America, from 1.6% in a Haitian hospital to 40% in Chile [2]. CS rates in Tanzania range from 21.4%-31.8% [3,4].

SSI is a common postoperative complication and is associated with significant morbidity and mortality [5-7]. The occurrence of an SSI following a CS reported in literature ranges from 0.3% in Turkey [7] to 24% [4] in Tanzania. The development of post CS infection depends on a complex interplay of many factors including: wound class, immune status, maternal age, hypertensive disorders, ASA classification, number of vaginal examinations, the virulence of the microorganisms, maternal weight, surgical techniques and premature rupture of membrane [8,9]. Pathogens that infect CS surgical wounds can be part of the patient’s normal flora (endogenous source), originate from the skin, vaginal and peritoneal cavities, or can be acquired from the hospital environment, other infected patients, and surgeons (exogenous source) [10-12].

The occurrence, risk factors and distribution of the pathogens isolated from post CS SSIs at Bugando Medical Centre are not known. Moreover, there is no evidence-based data to support the preoperative and postoperative care protocols at the centre. Therefore this study was carried out to determine the incidence, susceptibility pattern of the isolates and factors associated with SSI among pregnant women undergoing CS at Bugando Medical Centre. The results of this study are necessary to develop an evidence-based treatment protocol for post CS SSIs in this clinical setting and other settings with similar problems.

## Methods and materials

### Study design

This was a prospective cohort study to determine the incidence, predictors and patterns of surgical site infections among patients undergoing a CS at the Bugando Medical Centre(BMC) between October 2011 and February 2012. At BMC, no routine operation theatre (OT) and sterile services check up; however OT is undergoing major cleanliness once per week and whenever dirty case has been operated. All autoclaves are monitored using chemical and physical indicators daily.

### Inclusion and exclusion criteria

All patients who delivered by CS at Bugando Medical Centre and consented for the study were included in the study. Patients who failed to provide information and those who died during the procedure or immediately after CS were excluded from the study.

### Sampling procedure and sample size

Patients were serially enrolled until the sample size was reached. The sample size was calculated using the Schlesselman formula of the cohort study [13]. The occurrence rate used was the rate of SSIs among patients with obesity and those without obesity [14]. The minimum sample size obtained was 248, but the study enrolled 345 pregnant women.

### Data collection

Data was collected using a standardized questionnaire. The data collected included patient, pre- /intra- and postoperative information such as demographic data, wound characteristics and laboratory investigations. Data collected included patient characteristics such as age, parity, co-morbidities such as BMI, DM, and hypertension; pre-operative data such as labour characteristics, preoperative hospital stay and preoperative skin preparation. Intra-operative data collected were type of caesarean section, duration of operation, extent of surgical wound contamination, surgical techniques, blood loss volume, antibiotic prophylaxis and type of anaesthesia used.

Surgical wounds were inspected at the time of dressing on day 3 then on day 7 when stitches are removed and thereafter telephone calls were used in every 4 days to enquire for development of SSI and all patients were re-examined at day 30 post operatively Surgical site infection was defined as per Centres for Disease Control and Prevention’s National Nosocomial Infections Surveillance System as described below [15]. Superficial incisional SSI was defined as infection which occurs within 30 days after the operation and infection involves only skin or subcutaneous tissue of the incision and at least one of the following: Purulent drainage, with or without laboratory confirmation, from the superficial incision or organisms isolated from an aseptically obtained culture of fluid or tissue from the superficial incision; or at least one of the following signs or symptoms of infection: pain or tenderness, localized swelling, redness, or heat and superficial incision is deliberately opened by surgeon, unless incision is culture-negative, OR diagnosis of superficial incisional surgical site infection (SSI) by surgeon or attending physician.

Deep incisional SSI was defined as infection which occurs within 30 days after the operation and infection involves deep soft tissue (e.g. Fascial and muscle layers) of the incision and at least one of the following: Purulent drainage from the deep incision but not from the organ/space component of the surgical site, OR a deep incision spontaneously dehisces or is deliberately opened by a surgeon when the patient has at least one of the following signs or symptoms: fever (>38C), localized pain, or tenderness, unless site is culture-negative, OR an abscess or other evidence of infection involving the deep incision is found on direct examination, during re-operation, or by histopathologic or radiological examination, OR diagnosis of a deep incisional SSI by a surgeon or attending physician.

*Organ/space SSI* infection was defined as infection occurs within 30 days after the operation *and* infection involves any part of the anatomy (e.g. organs or spaces), other than the incision, which was opened or manipulated during an operation and at least *one* of the following: Purulent drainage from a drain that is placed through a stab wound into the organ/space *OR* organisms isolated from an aseptically obtained culture of fluid or tissue in the organ/space *OR* an abscess or other evidence of infection involving the organ/space that is found on direct examination, during re-operation, or by histopathologic or radiological examination *OR* diagnosis of an organ/space SSI by a surgeon or attending physician.

### Specimen collection and laboratory procedures

Specimens were taken from all patients who clinically were suspected to have surgical site infections. Before sample collection surrounding skin was cleaned with 70% alcohol (Aldrich Sigma; Nairobi). Exudates were obtained from the open discharging wounds with a sterile cotton swab or with a syringe [16]. Swabs or aspirates were transported to the laboratory for processing immediately once obtained.

All specimens were processed in accordance with the standard operating procedures of the laboratory. Briefly specimens were inoculated on Blood agar and Mackonkey agar (Oxoid; UK) and incubated aerobically for 24–48 hours. Identification of bacteria was done using conventional physiological and biochemical methods. Biochemical and physiological methods included; gram stain, catalase reaction, coagulase reaction, haemolytic activity on 5% sheep blood agar plate, hippurate hydrolysis and CAMP test for Gram positive bacteria while for gram negative; colonies morphology on blood agar and MacConkey agar, triple sugar iron (TSI) reaction, indole, motility, citrate, urease and hydrogen sulphide production (Oxoid, UK) were used [17,18]. Antimicrobial susceptibility of isolates was determined using disk diffusion method according to Clinical Laboratory standard Institute; for gram positive disks tested included penicillin G (10 IU), ampicillin (10 μg), clindamycin (15 μg), erythromycin(15 μg), vancomycin(30 μg), cefazolin (30 μg) and ciprofloxacin (5 μg) (Oxoid, UK). For gram negative disks tested included ampicillin (10 μg), amoxicillin/clavunic acid (20/10 μg), ciprofloxacin (5 μg), gentamicin (10 μg), ceftriaxone (30 μg), ceftazidime (30 μg) and meropenem (10 μg) (Oxoid, UK). MRSA was identified using cefoxitin disc (30 μg) and Oxacillin disc (1 μg) and interpreted as per CLSI [19].

### Data analysis

Data collected was entered into a computer using Epidata version 3.1 (CDC, Atlanta, USA) and analyzed using STATA version 11 (College Station, Texas, USA). Data was summarized in the form of proportions and frequent tables for categorical variables. Medians with standard deviation or medians with interquartile range were used to summarize continuous variables. A chi-square test was performed to test for significant associations between the predictor and outcome variables within the categorical variables and for continuous variables a Student t-test was performed. Hazard ratio (HR) with 95% confidence interval (CI) was calculated to test for the strength of association between predictor variables and SSIs using univariate analysis followed by multivariate Cox regression analyses for all predictors found to be significant on the univariate analysis. Significant association was defined as a p-value of less than 0.05.

### Ethical clearance

Catholic University of Health and Allied Sciences /Bugando Medical Centre review board cleared the study and informed consents were obtained from patients.

### Study limitations

Twenty six percent of the patients who developed an SSI after discharge did not return for specimen collection. No anaerobic culture was done and lack of antibiotic policy may have affected the SSI rate.

## Results

### Patients’ characteristics

**Figure 1:**
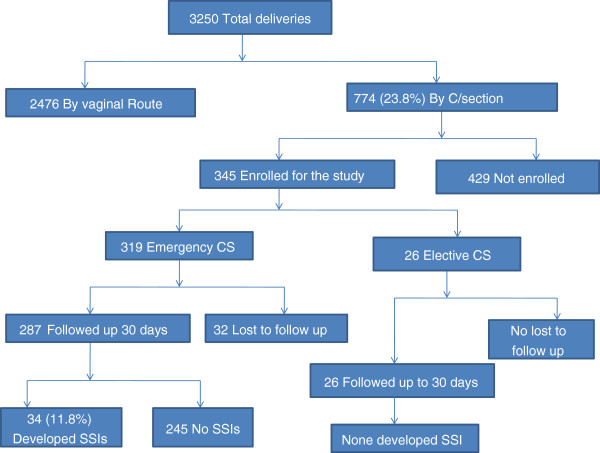
Flow chart of patient recruitment and follow up.

A total of 3250 deliveries were performed during the study period, 2476 (76.2%) by the vaginal route and 774 (23.8%) by CS (Figure 1). A total of 345 pregnant women who underwent CSs were enrolled and followed up for 30 days for development of SSI. Of these 345 CS; 319 (92.5%) were emergency procedures and 26 (7.5%) as elective procedures (Table 1).

**Table 1:** Clinical characteristics of patients undergoing caesarean sections, with and without subsequent surgical site infections

**Characteristic**	**n (%)**
**Age in years**	
<20	30 (8.7)
20-34	273 (79.1)
≥ 35	42 (12.2)
**Parity**	
Nulliparous	127 (36.8)
1-3	180 (52.2)
≥4	38 (11.0)
**Gestational age in weeks**	
˂ 37	68 (20.2)
≥37	268 (79.8)
**Mode of admission**	
Self	289 (83.8)
Referral	56 (16.2)
**Onset of labor**	
Spontaneous	260 (75.4)
Induced	21 (6.1)
No labor	64 (18.5
**Type of skin incision**	
Vertical	259 (75.1)
Transverse	86 (24.9)
**Type of caesarean section**	
Elective	26 (7.5)
Emergency	319 (92.5)
**Type of skin incision**	
Transverse	86 (24.9)
Vertical	259 (75.1)
**Number of prior caesarean sections**	
0	214 (62.0)
1	88 (25.5)
2	31 (9.0)
3	10 (2.9)
4	2 (0.6)
**HIV status**	
Positive	22 (6.4)
Negative	218 (63.2)
Unknown	105 (30.4)
**Body mass index (N = 345)**	
Underweight (<18.5)	1 (0.3)
Normal weight (18.5-24.9)	46 (13.3)
Overweight (25–29.9)	162 (45.0)
Obesity ≥30	136 (39.4)

The age of the women ranged from 14 to 44 years with a mean of 26.8 ± 5.8 years. The majority of women (273, 79.13%) were between 20 to 34 years of age and 218 (63.3%) were multiparas. The gestational age at caesarean delivery ranged between 28–44 weeks and a total of 268 neonates (79.8%) were delivered at term. Scars as indication of previous CS were found in 106 (30.7%) patients (Table 2).

**Table 2:** Indications for caesarean section

**Indication**	**N (%)**
Previous caesarean delivery	106 (30.7)
Fetal distress	54 (15.7)
Prolonged labor	48 (13.9)
Malpresentation	32 (9.3)
Obstructed labor	28 (8.1)
Hypertensive disorders of pregnancy	30 (8.7)
Antepartum hemorrhage	9 (2.6)
Premature rupture of membranes	7 (2.0)
Others*	31 (9.0)
**Total**	**345 (100.00)**

*Included maternal distress, maternal request, big baby, twin with Malpresentation, ruptured uterus, cord accident and failed induction of labor.

### Magnitude and burden of surgical site infections

During the study period, 34 patients developed SSIs resulting in an overall cumulative incidence of 34/312 (10.9%) and an incidence rate of 37.5 per 10,000 people/day (95% CI, 26.8-52.4). Types of SSI found were superficial SSI (21, 61.8%), organ space (8, 23.5%) and deep SSI (5, 14.7%). All SSIs occurred among patients who had emergency CSs. The rate of SSI was 12/202(5.9%) for clean wounds and 22/143(29.4%) for clean-contaminated wounds (p = 0.028 Fischer exact test). Most SSIs occurred among patients who had a vertical skin incision (31/43; 91.2%).

All SSIs occurred between the 3rd and 22nd day postoperatively with a median time of occurrence of 7 days post operation (IQR 6–9 days). Patients with a SSI had longer hospital stays than those without a SSI (12.7 ± 6.9 vs. 4 ± 1.7; *P* < 0.0001). The majority of patients with SSIs (n = 23/ 67.6%) were readmitted and one patient with an organ/space SSI died 2 days post relaparotomy due to septicemia.

### Predictors of surgical site infections

Univariate analysis indicated that post caesarean SSI occurred significantly more often among women with: hypertensive disorders of pregnancy (HR = 2.2; 95% CI = 1.1-4.7; *p =* 0.031), higher ASA physical status class (HR = 2.7; 95% CI = 1.3-5.5; *p =* 0.005), prolonged duration of labor (HR = 3.0; 95% CI = 1.5-6.0; *p =* 0.002), rupture of membranes prior to surgery lasting 8 hours or longer,(HR = 2.7; 95% CI = 1.3-5.8; *p =* 0.011), 3 or more vaginal examinations (HR = 3.3; 95% CI = 1.7-6.5; *p =* 0.001), higher wound class prior to surgery (HR = 2.7; 95% CI = 1.4-5.5; *P =* 0.005), vertical skin incision (HR = 3.6; 95% CI = 1.1-11.8; *p =* 0.034), prolonged duration of operation (a surgical procedure lasting longer than 1 hour) (HR = 2.4; 95% CI = 1.1-5.0; *p =* 0.020), and a procedure performed by an intern or junior doctor (HR = 2.8; 95% CI = 1.3-6.1; *p =* 0.012) (Tables 3 and 4).

**Table 3:** Univariate Analysis of preoperative risk factors for surgical site infection (SSI) in patients who underwent caesarean sections

	**Surgical site infection (SSI)**			
**Predictive factors**	**Yes n (%)**	**No n (%)**	**HR (95% CI)**	**p - value**
Age in years				
>30	4 (4.4)	86 (95.60	1.0	
≤30	30 (11.8)	225 (88.2)	2.8 (1.0-7.9)	0.055
Parity				
Multiparas	19 (8.7)	119 (91.3)	1.0	
Nulliparous	15 (11.8)	112 (88.2)	1.3 (0.7-2.6)	0.393
BMI‡				
None obese	30 (9.6)	282 (90.4)	1.0	
Obese	4 (12.1)	29 (87.9)	1.3 (0.4-3.6)	0.647
HIV status				
Negative	23 (10.6)	195 (89.5)	1.0	
Positive	4 (18.2)	18 (81.8)	1.8 (0.6-5.1)	0.295
Hypertension				
No	24 (8.3)	265 (91.7)	1.0	
Yes	10 (17.9)	46 (82.1)	2.2 (1.1-4.7)	0.031
ASA score*				
1 or 2	22 (7.8)	260 (92.2)	1.0	
≥3	12 (19.1)	51 (80.9)	2.7(1.3-5.5)	0.005
Hemoglobin level in g/dl				
>11	24 (10.0)	217 (90.0)	1.0	
<11	10 (10.4)	86 (89.6)	1.0 (0.5-2.1)	0.960
Duration of ROM† in hrs				
≤8	25 (8.3)	277 (91.7)	1.0	
>8	9 (20.9)	34 (79.1)	2.7 (1.3-5.8)	0.011
Number of vaginal exams				
≤3	20 (7.1)	260 (92.9)	1.0	
>4	14 (21.5)	51 (78.5)	3.3 (1.7-6.5)	0.001
Duration of labor in hrs				
≤12	20 (7.3)	256 (92.7)	1.0	
>12	14 (20.3)	55 (79.7)	3.0 (1.5-6.0)	0.002
Preop length of stays in hrs				
≤12	17 (7.8)	200 (92.2)	1.0	
>12	17 (13.3)	111 (86.7)	1.7 (0.9- 3.4)	0.116

*ASA = American Society of Anesthesiologists †ROM = rupture of membranes.

‡ BMI = Body mass index in kg/M^2^.

**Table 4:** Univariate analysis of intraoperative risk factors for surgical site infection (SSI) among patients who underwent caesarean sections

	**Surgical site infection (SSI)**			
**Predictive factors**	**Yes n (%)**	**No n (%)**	**HR (95% CI)**	**p - value**
Wound class				
Clean or clean-contaminated	12 (5.9)	190 (94.1)	1.0	
Contaminated or dirty	22 (15.4)	121 (84.6)	2.7 (1.4-5.5)	0.005
Type of anaesthesia used				
Regional	32 (9.7)	298 (90.30	1.0	
General	2 (13.3)	13 (86.7)	1.5 (0.4-6.5)	0.551
Type of skin incision				
Transverse	3 (3.5)	83 (96.5)	1.0	
Vertical	31 (12.0)	228 (88.0)	3.6 (1.1-11.8)	0.034
Duration of procedure in mins				
≤60	24 (8.3)	265 (91.7)	1.0	
>60	10 (17.9)	46 (82.1)	2.4 (1.1-5.0)	0.020
Type of surgeon				
Senior*	26 (8.4)	282 (91.6)	1.0	
Junior**	8 (21.6)	29 (78.4)	2.8 (1.3-6.1)	0.012
Number of people in theatre				
≤5	8 (13.8)	50 (86.2)	1.0	
>5	26 (9.1)	260 (90.9)	0.7 (0.3- 1.4)	0.289
Estimated blood loss in mls				
≤500	25 (9.3)	245 (90.7)	1.0	
>500	9 (12.0)	66 (88.0)	1.4 (0.6-2.9)	0.436
Antibiotic used				
Non-ampicillin regimen	14 (8.2)	156 (91.8)	1.0	
Ampicillin regimen	20 (11.5)	154 (88.5)	1.4 (0.7- 2.8)	0.304
Duration of antibiotic course				
Single dose	7 (8.5)	75 (91.5)	0.8 (0.4-1.8)	0.608
Multiple doses	27 (10.3)	235 (89.7)	1.0	

*Abbreviations: HR* hazard ratio, *CI* confidence interval.

*Senior doctor included postgraduate student, registered medical doctor and specialist.

**Junior surgeon defined an intern doctor.

Approximately 92% of CSs in this study were emergency procedures and all SSIs occurred in this group. However the following factors had no effect on the occurrence of SSI; age, gravidity, parity, gestational age at delivery, obesity, diabetes mellitus, anemia, preoperative length of stay, HIV infection, type of anaesthesia used, number of people in theatre, timing of antibiotic prophylaxis and the amount of blood loss intraoperatively.

Multivariate analysis did not include factors with high collinearity such as ASA and duration of rupture of membrane. The independent risk factors for post caesarean SSI were identified in this study by multivariate Cox regression analysis were hypertensive disorders of pregnancy (HR = 2.9; 95% CI, 1.4-6.4; *p =* 0.006); contaminated wound (HR = 2.5; 95% CI,1.2-5.1; *p =* 0.016), multiple vaginal examinations (HR = 2.6; 95% CI,1.3-5.3; *p =* 0.008), prolonged duration of operation (HR = 2.3; 95% CI,1.1-4.8; *p =* 0.001) and an operation performed by an intern or junior doctor (HR = 4.2; 95% CI,1.8-9.5; *p =* 0.030) (Table 5).

**Table 5:** Multivariate Model of risk factors for surgical site infection (SSI) in patients who underwent caesarean sections

**Independent risk factors**	**HR (95% CI)**	***P* - value**
Hypertensive disorders of pregnancy	2.9 (1.4-6.4)	0.006
Contaminated wound	2.5 (1.2-5.1)	0.016
Multiple vaginal examinations	2.6 (1.3-5.3)	0.008
Operation done by Intern doctor	4.2 (1.8-9.5)	0.001
Severe anaemia (Hb <7 g/dl)	3.8 (1.2-12.4)	0.028
Duration of procedure >60 minutes	2.3 (1.1-4.8)	0.030

HR = hazard ratio, CI = confidence.

In this study, the risk of developing a SSI increased significantly from a HR of 2.9 for patients with a National Nosocomial Infections Surveillance System (NNIS) risk index of 1 to a HR of 9.1 in patients with a NNIS System risk index of 2.

Almost all (344, 99.7%) women received antibiotics as prophylaxis with different timings of administration either before or after skin incision. No specific policy was followed, and the choice of antibiotics used was based on indication of CS and surgeons preference. The antibiotics given could be divided into ampicillin based regimens (170/344) and non-ampicillin based regimen combinations (174/344). A higher SSI rate was observed among patients which received the ampicillin based regimen (11.5%) compared with those which received non-ampicillin based regimen (8.2%, p = 0.304, HR 1.4).

### Bacterial isolates and susceptibility pattern

Pus swabs for aerobic culture and sensitivity were collected for 25 (73.5%) of the clinically suspected postoperative infection cases. Of 25 aerobic cultures, 18 (72.0%) were culture positive and 4 had significant polymicrobial infections, resulting in a total of 22 bacterial isolates.

Among the etiological agents isolated, 14 (63.6%) were gram-negative bacilli. *Staphylococcus aureus* was the most common organism (6, 27.3%). Of *Staphylococcus aureus*, 5 (83.3%) were methicillin sensitive *Staphylococcus aureus* (MSSA) and 1 (16.7%) was methicillin resistant *Staphylococcus aureus* (MRSA). Other isolates include *Klebsiella spp* (5, 22.7%), *Escherichia coli* (3, 13.6%), *Acinetobacter spp* (2, 9.1%), *Pseudomonas spp* (2, 9.1%), *Proteus spp* (1, 4.5%), *Enterobacter spp* (1, 4.5%), *Micrococcus spp* (1, 4.5%) and coagulase negative *Staphylococcus spp* (1, 4.5%).

The majority of gram-positive bacteria isolated were 100% sensitive to vancomycin and imipenem and 85% sensitive to cefazolin. Most of the enteric gram-negative bacteria were highly resistant to ampicillin (100%), amoxicillin/clavulanate (93%), sulphamethaxazole/trimethoprim (78.5%), fosfomycin (70%), tetracycline (70%) and gentamicin (35.7%). They were 100%, 85.7%, 85.7% and 78% sensitive to meropenem, ceftazidime, ciprofloxacin and ceftriaxone respectively. A total of 2 (13%) of the enteric gram-negative bacteria were found to produce extended spectrum beta lactamases (ESBL). Out of 8 organ infections, 5 (62.5%) were due to gram-negative enteric bacteria and 29.4% (5/17) of incisional SSIs were caused by *Staphylococcus aureus.*

## Discussion

### Magnitude of post cesarean section surgical site infection

As reported in many other studies in the developing countries [20,21],the incidence of SSIs in the present study was higher than in developed countries. This could be explained by standard of hygiene practiced in developed countries. In this study the incidence of SSIs following CS was 10.9%, which is lower than previously reported in Tanzania. At study at St. Francis Ifakara Hospital reported a cumulative incidence rate of 24% [20], which could be explained by lack of infection prevention policies in Ifakara during their study. The incidence of SSI following CSs was found to vary widely depending on the surveillance methods used to identify infections, criteria used to define SSI, postoperative hospital stays, antibiotic prophylaxis and the patient population [20,21]. In the present study the majority of patients had clean contaminated wounds. Previous studies have reported that patients with contaminated wounds had a 2.7 fold increased risk of developing a SSI than those with clean contaminated wounds [22-24]. The majority of post CS SSIs found in this study were superficial infections followed by deep tissue infections, similar to the findings were observed in previous studies [25]. Also reported previously [26], the incidence of deep tissue infections in this study was 14.7% and that of organ space infection was 5%.

Enteric gram-negative bacteria have previously been reported to be associated with severe SSI [27,28] . In the present study 62.5% of organ space infections were caused by gram negative enteric bacteria. This could be explained by the synergistic effect of facultative anaerobes and anaerobic infections.

### Predictors of post cesarean section surgical site infection

Various risk factors have been found to predict post caesarean SSI [8,9,26]. One patient factor is younger maternal age [14,29]; in the present study, an association was recorded between maternal age and SSI, suggesting that women aged 30 years or younger were more likely to have a SSI than those older than 30 years. Despite this trend, the association was not proven to be statistically significant (p = 0.055). In accordance with previous studies [24,30], hypertensive disorder of pregnancy was a predictor of SSI when analysed by both univariate (p = 0.031) and multivariate analysis (p = 0.006). This link could be explained by the chronic alteration of peripheral blood supply due to the increased vascular resistance.

In contrast to previous studies [14,29], the association of obesity and SSI was not statistically significant (p = 0.647, HR 1.3). Obesity has previously been reported to predict SSI via various possible factors, including the relative avascularity of adipose tissue. Another factor may be technical difficulties of handling adipose tissue which can result in more traumas to the anterior abdominal wall, or difficulty in obliterating dead space in the fat-tissue of the abdominal wall. The lack of significant association in this study may be due to the fact the Body Mass Index of patients in this study was measured during labour rather than before pregnancy.

ASA physical status classification score of 3 or more was significantly found to predict SSI (p = 0.005), in agreement with previously reported findings [31,32]. Prolonged labor (≥12 hrs), prolonged rupture of membranes (≥8 hrs) and multiple vaginal examinations (≥4 times) were significant predictors of SSI in this study, in agreement with previously obtained results [4,8,26]. Normally during pregnancy, cervical mucus plug, fetal membranes and amniotic fluid all serve as barriers to infection. However when the membrane is ruptured, this protective effect is gradually reduced over time as amniotic fluid becomes no longer sterile. It is thought that the non-sterile amniotic fluid may act as a transport medium by which bacteria come into contact with the uterine and skin incisions leading to chorioamnionitis and its sequelae.

Another important risk factor for SSI is the absence or delay of antibiotic prophylaxis. In this study there was no standard policy of antibiotic prescription. The choice of antibiotic administered depends on the surgeon and indication of CS. The antibiotics given in this study could be divided into two groups; an ampicillin based regimen and a non-ampicillin based regimen. The timing of antibiotic administration was not consistent. In contrast to previous studies [33,34] no significant difference was observed regarding type of antibiotic prophylaxis and SSI.

In this study emergency procedures were a strong predictor of SSI. This finding could be due to sampling bias because approximately 92.5% of CSs were done on an emergence basis. Vertical incision was significantly found to predict SSI; women with vertical skin incisions had a 3.6 fold risk of developing a SSI compared to those with transverse skin incision, and this has been observed before [29]. Prolonged operating times of longer than one hour has previously been found to be associated with SSI due to increased duration to exposure to microorganisms in the operating theatre [16,26,35] and this was confirmed by this study. The experience of surgeons performing the CS was also a critical determinant of SSI. Excellent surgical technique such as effective homeostasis while preserving adequate blood supply, preventing hypothermia, gently handling tissues, avoiding inadvertent entries into a hollow viscus, choice of appropriate suture material, eradicating dead space, and appropriately managing the postoperative incision are widely believed to reduce the risk of SSI [36]. In the present study, operations performed by an intern or junior surgeon increased the risk for SSI 4 fold, as shown by multivariate analysis. This could be explained by the fact that majority of junior surgeons made vertical incisions, had less experience in handling the tissue and control of blood loss, and the procedures were prolonged for more than 1 hour.

### Bacterial infection pattern

The microbial etiology of post CS SSIs has been shown to be diverse, being associated with both vaginal microorganisms such as *Escherichia coli*, group B streptococcus (GBS) and *Enterococcus spp,* or with nasopharyngeal flora such as *Staphylococcus aureus* or skin flora such *Staphylococcus epidermidis*[16]. *Staphylococcus aureus* has been found to be the most common cause of SSI post CS [17,37]. Other organisms such as *Escherichia coli, Klebsiella* spp., *Pseudomonas aeruginosa*, *Enterobacter* spp., *Proteus* spp. and *Enterococcus* spp., show a variable distribution pattern [16]. A similar pattern has been observed in this study; *Staphylococcus aureus* was the most common isolate taken from superficial skin infections followed by *Klebsiella pneumoniae* in deep and organ infections. As described in other studies most of *Staphylococcus aureus* isolated from SSIs were MSSA and only 1 isolate (16.7%) was found to be MRSA. The prevalence of MRSA is similar to the previous study carried out in the same hospital in 2009 [20]. In the present study the majority (37.5%) of *Staphylococcus aureus* isolates were resistant to ampicillin, co-trimoxazole and erythromycin; similar to the findings of Fantahamu *et al.*[38]. The rate of resistance to these antibiotics was higher than the rate of resistance to ciprofloxacin and gentamicin, which could be explained by self prescription to these antibiotics in the community.

As reported previously, the majority of gram-negative enteric bacteria were highly resistant to ampicillin, amoxycillin/clavulanate, tetracycline and co-trimoxazole [11]. A total of 10-22% of gram-negative enteric bacteria was found to be resistant to third generation cephalosporins, in accordance with previous findings [11]. In this study only one patient with organ space infection died, and this was due to a polymicrobial infection of *Staphylococcus aureus* and *Klebsiella pneumoniae* which was an ESBL producer. Patients with a SSI had prolonged hospitalization compared with those without a SSI (12.7 vs 4.1 days), similar to the results observed by Killian *et al.*[26].

## Conclusion

Hypertensive disorders of pregnancy, contaminated wound, multiple vaginal examinations, operations carried out by an intern or junior doctor and prolonged duration of the surgical procedure (longer than 60 minutes) have been found to be independent factors which increase the risk of SSI at Bugando Medical Centre. Identifying high-risk patients who require intensive postoperative care is critical in order to reduce the incidence of SSIs. This can be achieved if independent risk factors for SSI are well understood within the given clinical setting.

## Competing interests

The authors declare that they have no competing interests.

## Authors’ contributions

FJM; participated in collecting specimens, collecting clinical data and follow up of the patients and data analysis, SEM; participated in the design and execution of the work, performed microbiological procedures, data analysis, interpretation of data and preparation of the manuscript, MM: Perform data analysis and writing the manuscript; BRK; participated in data analysis, BG; designed the study, collected clinical data and participated in manuscript writing, CI; Designed the study, participated in the interpretation of the data and manuscript writing. All authors have read and approved the final manuscript.
